# Middle East Respiratory Syndrome Coronavirus (MERS-CoV) origin and animal reservoir

**DOI:** 10.1186/s12985-016-0544-0

**Published:** 2016-06-03

**Authors:** Hamzah A. Mohd, Jaffar A. Al-Tawfiq, Ziad A. Memish

**Affiliations:** Saudi Ministry of Health, Riyadh, Saudi Arabia; Johns Hopkins Aramco Healthcare, Dhahran, Saudi Arabia; Indiana University School of Medicine, Indianapolis, IN USA; College of Medicine, Alfaisal University, P.O. Box 54146, Riyadh, 11514 Kingdom of Saudi Arabia

**Keywords:** MERS-CoV, Coronavirus, Middle East, Animal, Dromedary, Camels, Bats, Middle East Respiratory Syndrome

## Abstract

Middle East Respiratory Syndrome-Coronavirus (MERS-CoV) is a novel coronavirus discovered in 2012 and is responsible for acute respiratory syndrome in humans. Though not confirmed yet, multiple surveillance and phylogenetic studies suggest a bat origin. The disease is heavily endemic in dromedary camel populations of East Africa and the Middle East. It is unclear as to when the virus was introduced to dromedary camels, but data from studies that investigated stored dromedary camel sera and geographical distribution of involved dromedary camel populations suggested that the virus was present in dromedary camels several decades ago. Though bats and alpacas can serve as potential reservoirs for MERS-CoV, dromedary camels seem to be the only animal host responsible for the spill over human infections.

## Background

A novel coronavirus was isolated from a Saudi Arabian patient with severe acute respiratory syndrome in June 2012 and the virus was later named Middle East Respiratory Syndrome - Coronavirus (MERS-CoV) [1]. Since then, multiple outbreaks have been reported in or been epidemiologically linked to the Arabian Peninsula. Up to April 18, 2016, the Saudi authorities reported a total of 1386 cases of which 587 died [2]. With a high mortality rate, lack of antiviral treatment, or preventive vaccine, MERS-CoV remains a major public concern.

The first human coronavirus was cultivated in 1965 on human ciliated embryonal tracheal cells [3]. Literature about human coronaviruses was limited up until the Severe Acute Respiratory Syndrome Coronavirus (SARS-CoV) outbreak in 2002. The interest in coronaviruses reemerged as a hot topic after MERS-CoV outbreaks. Coronaviruses are enveloped, positive stranded RNA viruses classified as a family within the Nidovirales order. They have a crown appearance under the electron microscopy due to the spike protein on the surface. The coronavirus subfamily is classified into genera: alpha, beta, gama, and delta. Human coronaviruses belong to alpha, and beta coronaviruses [4].

MERS-CoV is a lineage C betacoronavirus with a genotype that is very closely related to bat coronaviruses from the same lineage, such as BtCoV-HKU4 and BtCoV-HKU5, though its evolutionary pathway is still unclear [5].

A limited number of coronaviruses is known to cause human disease. Most of known coronaviruses infect and circulate in animals, mainly bats. So when it comes to a new novel coronavirus with limited geographic distribution, one would think of a zoonotic disease with animal reservoir. This proved to be true with SARS and seems to be the case with MERS-CoV. The origin of the virus and the extent of its involvement in both human and animal populations remain hot topics that are being explored with phylogenicity and surveillance studies. In this review we summarize the current evidence about MERS-CoV origin and animal reservoir.

### MERS-CoV origin

It is believed that MERS-CoV, like many other coronaviruses, originated in bats. This is based on the isolation of other lineage C beta-coronaviruses that are very closely related to MERS-CoV on phylogenetic analysis [6, 7].

A large screening study for beta-coronaviruses from 5030 bats’ fecal specimens was conducted between 2009 and 2011. In that study 4758 bats of ten different species from Ghana, and 272 Pipistrellus bats from four European countries were included [8]. Reverse Transcription Polymerase Chain Reaction (RT-PCR) was used to detect coronavirus RNA. Of the ten bat species tested in Ghana, only *Nycteris gambiensis* was found to carry 2c beta-coronavirus. A round one fourth of the 185 Nycteris bats tested positive; this accounted for 1 % of whole tested bat’s population from Ghana. Of the Pipistrellus bat species tested in Europe, 40 out of 272 (14.7 %) carried 2c beta-coronavirus. Both 2c beta-coronaviruses isolated are genetically very closely related to MERS-CoV. This relatedness indicates that MERS-CoV likely originated from bats [8]. Though the study had a good sample size and screened different geographical areas, none of those areas is known for MERS-CoV infection in humans or domestic animals.

A report from South Africa identified a bat derived coronavirus that has a very close phylogenetic relationship to MERS-CoV [9]. The virus was isolated from a *Neoromicia* cf. *zuluensis* bat sampled in 2011 [9]. In Saudi Arabia, bat fecal samples collected in October of 2012 were tested for MERS-CoV RNA. A product obtained by PCR amplification of nucleic acid from a fecal pellet of a *Taphozous perforatus* bat captured in Bisha showed 100 % nucleotide match to the MERS-CoV cloned from an index case patient living in the same area [10]. Though it was a single 190-nucleotide segment, it does give a clue that MERS-CoV might be circulating in bats and that *Taphozous perforatus* (Egyptian tomb bat) might be a source of MERS-CoV.

A study of 821 bats captured in Egypt and Lebanon between February 2013 and April 2015 did not detect MERS-CoV in any of the bats sampled [11]. In that study three species: *Taphozous perforatus*, *Pipistrellus deserti*, and *Rousettus aegyptiacus*, were sampled from Egypt, and four species: *Rhinolophus hipposideros*, *Miniopterus schribersii*, *Rhinolophus ferrumequinum*, and *Rousettus aegyptiacus*, were sampled from Lebanon [11]. Serum samples, oral and rectal swabs were collected and tested from live bats. Homogenized lung and liver material was tested from 72 bats that died or were euthanized upon capture [11]. One limitation of this study is that most of the bats screened belong to one species, *R. aegyptiacus* (85 %), and limited numbers of the other species were tested, especially *T. perforatus* (10 %).

To further investigate the role of bats as a potential reservoir for MERS-CoV, an experimental study was conducted on Jamaican fruit bat (*Artibeus jamaicensis*). Ten bats were inoculated through intranasal and intraperitoneal routes with MERS-CoV [12]. All bats showed evidence of infection as they shed the virus from their respiratory and, to a lesser extent, intestinal tract, but none of the bats showed clinical signs of disease [12]. The ability of MERS-CoV to replicate in bats without clinical signs of disease indicates that they can serve as a reservoir for MERS-CoV.

Thus, MERS-CoV, or its immediate ancestor, could have likely originated in bats, and that bats can serve as an ideal reservoir for MERS-CoV. It is difficult to proof that bats are the direct source of human disease, based on the available bats’ screening studies. In addition, here is no clear direct contact between bats and humans, especially in Saudi Arabia where most of the cases are being diagnosed. A summary of important studies that screened bats for beta-coronaviruses and MERS-CoV is shown in Table 1.

**Table 1:** Summary of important studies that screened bats for beta-coronaviruses and MERS-CoV

Location	Year	Species	Number	Specimen	Virus	% Positive
Ghana [8]	2009–2011	*Nycteris cf. gambiensis*	185	Fecal	2c betacoronaviruses(closely related to MERS-CoV)	24.9 %
Ghana [8]	2009–2011	9 different species^a^	4573	Fecal		0 %
Europe [8] (Germany, Netherland, Romania, Ukraine)	2009–2011	*Pipistrellus kuhlii, P. nathusii, P. pipistrellus, P. pygmaeus*	272	Fecal		14.7 %
South Africa [9]	2011–2012	13 different species^b^	62	Fecal pellets	bat related-alphacoronaviruses betacoronavirus	6.4 % 1.6 %
Saudi Arabia [10]	2012	*Rhinopomahardwickii, R.microphyllum, Taphozous perforatus, P. kuhlii, Eptesicus bottae, Eidolon helvum, and Rosettus aegyptiacus*	96	Throat swab, serum, urine, rectal swab or fecal pellets	MERS-CoV	1 %
Saudi Arabia [10]	2013	*R.hardwickii, T.perforates, P.kuhlii*	14	Throat swabs, roost feces	MERS-CoV	0 %
Egypt [11]	2013–2015	*T. perforatus*	82	Serum/rectal (alive)	MERS-CoV	0 %
		*P. deserti*	31			
		*R. aegyptiacus*	257			
Lebanon [11]	2013–2015	*R. hipposideros*	4	Homogenized lung and liver material (if died or euthanized upon capture)		0 %
		*Miniopterus schribersii*	6			
		*R. ferrumequinm*	3			
		*R. aegyptiacus*	438			

^a^Coleura afra, Hipposiderosabae, H. cf. gigas, H. fuliginosus, H. jonesi, H. cf. ruber, Rhinolophus alcyone, R. landeri, Taphozous perforates

^b^ *Chaerephonpumilus, Mops condylurus, Tadaridaaegyptiaca, H.caffer, Miniopterus natalensis, Nycteristhebaica, R.clivosus, R. darlingi, Neoromicia capensis, N. nana, N. cf. zuluensis, Scotophilus viridis, Rousettus aegyptiacus*

## Animal reservoir

### Does MERS-CoV infect dromedary camels? Is there a role for dromedary camels in human disease?

The first evidence to link MERS-CoV to dromedary camels came from a serological study that investigated different animals: dromedary camels, cattle, sheep, goats and various other camelid species. MERS-CoV specific antibodies were only found in dromedary camels [13].

Another evidence to link MERS-CoV to dromedaries was found after two human cases of MERS-CoV infection, diagnosed in October of 2013, and were linked to a farm in Qatar [14]. In response, all the 14 dromedary camels on that farm were tested with RT-PCR. Eleven dromedary camels had positive nasal swabs for MERS-CoV. The nucleotide sequence of an ORF1a fragment and a 4 · 2 kb concatenated fragment of three dromedary camel samples were very similar to the sequence from the two human cases linked to that farm [14]. Another study from Saudi Arabia described a 43 year old male who owned nine dromedary camels and was in direct contact with them up until he was diagnosed with MERS-CoV infection in November 2013 [15]. Four of his dromedary camels were sick before his symptoms started. Cell cultures from a laboratory confirmed dromedary camel and the patient grew genetically identical MERS-CoV viruses [15].

In addition to providing a virological confirmation of MERS-CoV in dromedary camels, the last two studies indicated a potential cross infection between dromedary camels and humans and that the virus could be transmitted from dromedary camels to humans through close contact [14, 15]. A study that obtained the full genome of MERS-CoV from a dromedary camel in Qatar showed that MERS-CoVs from dromedary camel and humans are nearly identical [16].

A nation-wide cross-sectional serological study done in Saudi Arabia between December 1st 2012 and December 1st 2013 in which serum samples from slightly over ten thousand individuals, whom age and sex distribution largely matched the general population, were tested for MERS-CoV antibodies [17]. Positive results were confirmed in 15 individuals (0.15 %). Of note, seropositivity was significantly higher in dromedary camel exposed individuals compared to the rest of the study population; fifteen times higher in shepherds and twenty three times higher in slaughterhouse workers [17]. This study does not only support the theory of dromedary camel role in human infection but also indicates a higher prevalence of human seropositivity than expected, based on reported cases from Saudi Arabia.

In another study, 498 serum samples obtained from Qatar and Europe between 2013 and 2014 were tested for MERS-CoV antibodies. 294 samples were obtained from persons with occupational dromedary camel contact in Qatar; the remaining 204 samples were obtained from persons with no dromedary camel contacts from both Qatar and Europe. Around 7 % of those with dromedary camel contact tested positive for MERS-CoV antibodies, while none of the samples from persons with no dromedary camel contacts tested positive [18].

A case control study assessed differences in environmental exposure between thirty laboratory confirmed primary MERS-CoV cases and 116 controls. The study was conducted between March and November of 2014, and the cases represented 8 out of 13 regions in Saudi Arabia. The study concluded that direct dromedary camel exposure within two weeks of symptoms onset is independently associated with MERS-CoV infection [19].

So the evidence points to a close dromedary camel contact as a major risk for human infection. In order to better understand the ecology of MERS-CoV infection in dromedary camels, three adult dromedary camels were inoculated with a human isolate of MERS-CoV. Each dromedary camel was inoculated though intratracheal, intranasal and conjunctival routes. Transient, primarily upper respiratory tract infection developed in each of the three camels. Each dromedary camel shed large quantities of virus from the upper respiratory tract, evidenced by the presence of infectious viruses in nasal swab samples and, to a lesser extent, in oral samples. No infectious virus or viral RNA was detected in fecal, urine, serum or whole blood samples [20]. After authentication, the virus was recovered from respiratory tissues and some lymph nodes draining the respiratory system of one dromedary camel, but not in any other organs. These findings were consistent with the data from naturally infected dromedary camels [6, 15, 21, 22]. Some studies reported the detection of MERS-CoV virus from fecal specimen, though virus detection rates were far lower than in nasal swab specimens [22–24]. One study in particular reported detection of MERS-CoV RNA in the milk of five out of seven infected dromedary camels [23].

A vaccine expressing the MERS-CoV spike protein was shown to confer mucosal immunity in dromedary camels with evidence of serum neutralizing antibodies and significant reduction in excreted infectious virus and viral RNA transcripts in vaccinated animals [25].

Based on surveillance and epidemiological studies, it is obvious that MERS-CoV infects dromedary camels, which serve as a reservoir with spell over human infections through close dromedary camel contacts [13–15, 17–19, 24]. The exact routes of transmission are not very well understoodbut direct contacts with Dromedary camels or the handling of surfaces or objects contaminated with dromedary camels’ respiratory or fecal material may as well pose a risk for infection.

### What is the extent of MERS-CoV infection in dromedary camels?

Multiple surveillance studies explored the extent of MERS-CoV infection in dromedaries. Presence of specific MERS-CoV antibodies in dromedary camels’ sera was used as an indicator of previous exposure to the virus, while the presence of MERS-CoV RNA material in nasal secretions, usually identified through RT-PCR, indicated current infection and active viral shedding.

Serum samples from 303 dromedary camels from Saudi Arabia were screened in 2013 and found to have a high seropositivity of 72 % to MERS-CoV [21].

All serum samples from 50 dromedary camels in Oman were positive for MERS-CoV specific antibodies [13]. Similar results were reached from a larger study conducted in United Arab Emirates (UAE), where 500 dromedary camels’ sera screened in 2013 revealed 96 % seropositivity [26].

In Africa, a study assessed the geographic distribution of MERS-CoV among dromedaries by investigating serum samples from Nigeria, Tunisia, and Ethiopia. In Nigeria, serum samples collected between 2010 and 2011 from 358 adult dromedaries distributed over 4 provinces were tested, and 94 % were positive for MERS-CoV antibodies. In Tunisia, serum samples collected in 2009 and 2013 from 204 dromedaries distributed over 3 provinces were tested and 48.5 % were positive for MERS-CoV. In Ethiopia, 96.3 % of the serum samples collected between 2011 and 2013 from 188 dromedaries, distributed over 3 provinces, were positive for MERS-CoV antibodies [27].

Another study was conducted in Kano, Nigeria in January 2015. Nasal swabs and blood samples were collected from dromedary camels shortly after slaughter at a slaughterhouse. The samples were tested for the presence of MERS-CoV RNA using RT-PCR from nasal swabs. Sera were tested for the presence of specific MERS-CoV antibodies. Of the 132 dromedary camels screened with nasal swabs, 14 were found to carry the virus RNA (11 %). The overall seropositivity was 95 % [28].

A random group of 105 dromedary camels presented for slaughter in Qatar at two occasions in 2014 were sampled for MERS-CoV. Nasal, oral, bronchial and rectal swabs were tested for MERS-CoV. A high proportion (59 %) of them was actively shedding the virus at the time of slaughter. The percentage of positive samples was the highest for nasal samples, followed by oral swabs, fecal swabs, and bronchial swabs. Co-circulation of multiple MERS-CoV variants, evidenced by five different sequence types, demonstrates multiple virus introductions likely related to the flow of new dromedary camels from different origins [24].

Those findings indicate a widespread exposure to MERS-CoV in the dromedary camel populations of Africa and the Arabian Peninsula, to the extent that almost every adult dromedary camel got infected at some point in life.

### Do dromedary camels differ in their vulnerability to MERS-CoV infection based on age?

Two hundred and three samples from live dromedary camels in Saudi Arabia were collected in 2013 and found to have a high seropositivity (72 %) to MERS-CoV [21]. Seropositivity was higher among adults dromedary camels (two years and older) compared to juvenile dromedary camels (less than two years of age), 95 % vs. 55 % respectively [21]. Increasing seropositivity with age might be related to increased likelihood of exposure and subsequent infection over time.

In the same study, 202 dromedary camels’ nasal swabs were tested for the presence of MERS-CoV RNA material using RT-PCR; 25 % were positive. In other words, one fourth of the tested dromedary camel population was shedding the virus and was potentially infectious. Of those shedding the virus 71 % were juvenile and 29 % were adult dromedary camels older than two years [21]. The results indicate that juvenile dromedary camels might be at higher risk of contracting the virus. An explanation could be that they are naive to the virus and lack neutralizing antibodies.

### When was the virus introduced to dromedary camels?

In an attempt to investigate the time frame of MERS-CoV introduction to dromedary camel population, multiple studies screened stored serum samples. 100 % seropositivity was found in 151 dromedary camel serum samples obtained in 2003 from UAE dromedary camels [26]. Archived serum samples, obtained from dromedary camels in Saudi Arabia from1992 through 2010, were found to have a high seropositivity ranging from 72 % to 100 % [21]. 189 stored dromedary camel serum samples from Egypt, collected in 1997, and from Sudan and Somalia, collected between 1983 and 1984, were tested, and 81 % were found to have neutralizing antibodies to MERS-CoV [29]. Another study from Kenya showed similar results [30]. This implies that MERS-CoV has been heavily endemic in both the Middle East and East Africa’s dromedary camels for decades.

In addition to evaluating MERS-CoV distribution and the infection burden in dromedary camel populations, screening different geographic areas might also help predict when MERS-CoV was introduced to dromedary camels. Historically and up to the early twentieth century, both dromedary and bactrian camels played a vital role in carrying passengers and goods in North Africa, the Middle East, and East Asia. For the same reason, they were imported to Australia in the nineteenth century. After the invention of automobile and the use of trains, use and travel of dromedary and bactrian camels between countries was limited.

Serum samples collected from 105 dromedary camels living in the Canary Islands, a Spanish archipelago located just off the southern coast of Morocco, between 2012 and 2013 were tested, and 14 % were found to have antibodies against MERS-CoV [13]. This indicates that a small proportion of Canary Island dromedary camels were exposed to the virus at some point in time. This low percentage, compared to East Africa and the Arabian Peninsula, might be related to the isolated nature of the Canary Islands, as they are off the West African coast and are governed by Spain. Those two factors might limit the interaction of the island’s dromedary camels with those from other parts of Africa. This theory was later supported by another study conducted in early 2015. In this study, a representative sample of 170 dromedary camels on the Canary Islands was investigated; only 4.1 % were seropositive for MERS-CoV. All the seropositive dromedary camels were imported from Africa 20 or more years prior. This led to the conclusion that active infection and shedding didn’t take place on the Canary Islands [31].

In an attempt to screen feral camels in Australia, 307 dromedary camels’ sera from two different locations were sampled between December 2013 and June 2014. All tested negative for specific MERS-CoV antibodies [32].

In Kazakhstan 550 camels’ sera: 455 dormeday and 95 bactrian, camels with two humps (*Camelus bactrianus*), were screened for MERS-CoV between February and March 2015, and all tested negative [33]. Another surveillance study was conducted in southern Mongolia in November of 2014. In that study, 210 bactrian camels from 12 herds were screened. All samples tested negative, and the study concluded that MERS-CoV was not present in bactrian camels of the screened areas [34].

A serological and virological surveillance study of ten bactrian camel herds in three areas of the West of Inner Mongolia Autonomous Region (IMAR) was conducted in 2015. One hundred and ninety Bactrian camels were sampled; nasal swab and serum samples were collected from each Bactrian camel. All samples turned negative and the study concluded that there was no MERS-CoV circulating among Bactrian camels in the West IMAR [35].

Based on the above studies, it seems that MERS-CoV infection is limited to dromedary camel populations in Africa and the Middle East. This may indicate that dromedary camel infection could have been introduced at a point in time when the dromedary camel caravans between Asia, Middle East, and Africa were no longer active.

### Are there other domestic animal reservoirs for MERS-CoV?

In an attempt to investigate the possibility of other reservoirs, multiple domestic animal populations were screened. In a serological study, conducted in Saudi Arabia between 2010 and 2013, sera from 100 sheep, 45 goats, 50 cattle, and 240 chickens representing different geographical areas within the country were collected. All samples tested negative for MERS-CoV [36]. In another study, serum samples from 36 goats and 102 sheep from central Saudi Arabia tested negative for MERS-CoV antibodies as well [21]. In 2013 a similar study was conducted in Jordan. None of the 150 goats, 126 sheep and 91 cows tested was found positive for MERS-CoV antibodies [37]. Sera from European sheep, goats, and cattle had no evidence of MERS-CoV antibodies as well [13].

Equids were also screened for MERS-CoV antibodies. 192 adult horse samples from UAE and 861 samples from Spain (697 horses, 82 donkeys, and 82 mules) were screened. All were seronegative for MERS-CoV [38].

Dromedary camels seemed to be the only domestic animal reservoir for MERS-CoV up until a recent study conducted in Qatar in April of 2015 investigated the MERS-CoV infection status of 15 healthy alpacas (*Vicugna pacos*) in a herd of 20 that shared a barn complex with dromedaries. All tested alpacas were seropositive to MERS-CoV (100 %). Of note, MERS-CoV is endemic in Qatar’s dromedaries, and nine out of ten dromedary camels that shared the same barn complex were seropositive for MERS-CoV [39]. This indicates the susceptibility of alpacas for natural MERS-CoV infection and the potential for a new MERS-CoV animal reservoir. Of note, a previous study found no evidence for MERS-CoV infection in alpacas from regions where MERS-CoV is not endemic [13].

In another study 3 alpacas were experimentally infected through intranasal insulation of MERS-CoV viruses. All got infected and shed the virus; they also transmitted the infection to two out of three other alpacas that shared the same room. Similar to dromedary camels, infected alpacas didn’t develop fever, but unlike dromedary camels, none of the alpacas had any observable nasal discharge over the course of infection. All infected animals were able to mount neutralizing antibodies to MERS-CoV [40].

Those studies indicate that alpacas, similar to dromedary camels, can be infected and potentially serve as a reservoir.

## Conclusion

Our experience with MERS-CoV is of zoonotic nature, transmitted to humans from infected dromedary camels. The origin of MERS-CoV viral infection is not very well understood. It could have originated in bats and transmitted to dromedary camels at some unknown time in the past.

The virus seems to be well maintained in dromedaries, which serve as a reservoir with a spill over human infections. Sporadic human cases in areas where MERS-CoV is endemic in dromedary camels are likely to continue to happen.

The awareness of the disease and the easy access to a more developed health care system could explain the higher incidence of MERS-CoV diagnosis in Saudi Arabia compared to other countries in Africa where the disease is likely to be overlooked.

Larger scale serological screening of human populations in areas where MERS-CoV is endemic in dromedary camels should be considered. More extensive screening of bats in Saudi Arabia and East Africa, especially the Egyptian tomb bat, needs to be considered. Screening dromedary camel populations in Africa (Sahara desert and surrounding areas), and East Asia (Pakistan, Afghanistan, and Iran) will help better delineate the geographical distribution of dromedaries involvement.

Experimental MERS-CoV inoculation of other domestic animals will help define predisposed groups and should be considered so as to guide screening efforts for other potential reservoirs.

## Abbreviations

IMAR, Inner Mongolia Autonomous Region; MERS-CoV, Middle East Respiratory Syndrome - Coronavirus; RT-PCR, Reverse Transcription - Polymerase Chain Reaction; SARS-CoV, Severe Acute Respiratory Syndrome - Coronavirus; UAE, United Arab Emirates
